# Reducing Heart Failure Hospital Readmissions: A Systematic Review of Disease Management Programs

**DOI:** 10.14740/cr362w

**Published:** 2014-10-06

**Authors:** Janardhana Gorthi, Claire B. Hunter, Ayran N. Mooss, Venkata M. Alla, Daniel E. Hilleman

**Affiliations:** a The Creighton University Cardiac Center, Creighton University School of Medicine, Omaha, NE, USA

**Keywords:** Heart failure, Hospitalizations, Heart failure clinics, Telemanagement, Telemonitoring

## Abstract

The recent enactment of the Patient Protection and Affordable Care Act which established the federal Hospital Readmissions Reduction Program (HRRP) has accelerated efforts to develop heart failure (HF) disease management programs (DMPs) that reduce readmissions in patients hospitalized for HF. This systematic review identified randomized controlled trials of HF DMPs which included home care, outpatient clinic interventions, structured telephone support, and non-invasive and invasive telemonitoring. These different types of DMPs have been associated with conflicting results. No specific type of DMP has produced consistent benefit in reducing HF hospitalizations. Although probably effective at reducing readmissions, home visits and outpatient clinic interventions have substantial limitations including cost and accessibility. Telemanagement has the potential to reach a large number of patients at a reasonable cost. Structured telephone support follow-up has been shown to significantly reduce HF readmissions, but does not significantly reduce all-cause mortality or all-cause hospitalization. A meta-analysis of 11 non-invasive telemonitoring studies demonstrated significant reductions in all-cause mortality and HF hospitalizations. Invasive telemonitoring is a potentially effective means of reducing HF hospitalizations, but only one study using pulmonary artery pressure monitoring was able to demonstrate a reduction in HF hospitalizations. Other studies using invasive hemodynamic monitoring have failed to demonstrate changes in rates of readmission or mortality. The efficacy of HF DMPs is associated with inconsistent results. Our review should not be interpreted to indicate that HF DMPs are universally ineffective. Rather, our data suggest that one approach applied to a broad spectrum of different patient types may produce an erratic impact on readmissions and clinical outcomes. HF DMPs should include the flexibility to meet the individualized needs of specific patients.

## Introduction

The medical and financial burden of heart failure (HF) hospitalizations has led to a substantive body of research characterizing the timing and etiology of readmissions, identifying methods that predict readmission, and evaluating strategies that reduce readmissions. Findings from epidemiologic surveys of HF patients indicate that 30% of readmissions occur during the first 2 months after hospital discharge, 50% of readmissions occur within the last 2 months prior to death, and the remaining 20% of readmissions occur between these time periods [1, 2]. This pattern of readmissions has been referred to as the “three-phase terrain” of HF readmissions [3].

The Patient Protection and Affordable Care Act established the federal Hospital Readmissions Reduction Program (HRRP) through which Medicare payments to hospitals that have excess readmissions following an admission for HF, myocardial infarction, or pneumonia would be reduced [4]. The HRRP took effect on October 1, 2012 using claims data from July 2008 through June 2011. The CMS defines a readmission as any hospital admission that occurs within 30 days of a discharge from the same or other hospital [5]. Excess readmissions are calculated by comparing a hospital’s rate of readmission for an applicable condition against the national average for similar hospitals. For fiscal year 2013, excessive readmissions can result in a maximal loss of up to 1% of Medicare reimbursement for the coming year [6]. The HRRP is expanding in 2015 to include readmissions for chronic obstructive pulmonary disease, coronary artery bypass graft surgery, percutaneous coronary interventions, and other vascular interventions with penalties increasing to a maximum payment withholding of 3% [7].

The ability of HF disease management programs (DMPs) to routinely reduce all-cause hospital readmissions at 30 days has not been documented. Many HF DMPs have reported morbidity and/or mortality outcomes or have used different follow-up time points [3]. Many programs have not focused on clinical outcomes or reductions in unplanned healthcare contacts, but rather have evaluated the rate at which a DMP has been successful in changing the process of care in HF patients [8]. Since the inception of the HRRP, greater emphasis has been place on HF DMPs [9].

The purpose of the present systematic review was to critically evaluate all available studies meeting minimal inclusion criteria to define the efficacy of DMPs in reducing hospitalizations and/or mortality in patients with chronic HF. The recent HRRP initiative has provided substantial motivation to minimize hospital readmissions in patients discharged with a diagnosis of HF.

## Methods

Studies were identified using the guidelines defined by the Cochrane Handbook or Systemic Reviews and the Meta-analysis of Observational Studies in Epidemiology (MOOSE) [10, 11]. The on-line databases of PubMed (Medline), EBSCOHost, and the Cochrane Library were searched from January 1975 through August 2014 for studies reporting the outcomes of HF DMPs. The medical subject heading terms used in the search included HF DMPs, HF, hemodynamics, structured telephone support, telemonitoring, telemanagement, and implantable hemodynamic devices. A manual search of the bibliographies of the identified reports and reviews was also performed.

Only studies published in English were included in the analysis. Studies published only as abstracts were excluded. Only prospective, randomized studies including a minimum of 50 patients were included. Eligible studies had to report either hospitalizations (all-cause or heart failure specific) or mortality (all-cause or cardiovascular). Efficacy was based on study reported outcomes concerning hospital readmissions or mortality comparing the intervention and control or usual care treatment arms. Studies using pre- and post-disease management intervention analyses were excluded. Studies that were published as preliminary reports that were subsequently reported in a later publication with a larger sample size were not included in this analysis. In addition, studies reporting on patients with disease states other than HF which did not report outcomes for HF patients separately from other patient types were also excluded.

## Results

### In-home care interventions

A total of eight randomized controlled studies meeting eligibility criteria evaluating the efficacy of in-home visits as part of an HF DMP were identified (Table 1) [12-19]. One study included only one home visit and two others included only two home visits [13, 14, 17]. Six studies compared home visit interventions to usual care, one study compared home visits in addition to outpatient visits to usual care, and one study compared home visits with outpatient visits. Of the seven studies comparing home visits to usual care, three were associated with a significant improvement in the primary outcome [14-16]. None of the studies were able to demonstrate a significant reduction in all-cause mortality. One study was able to demonstrate a reduction in all-cause hospitalization which was driven by a reduction in HF hospitalizations [12]. Two studies significantly reduced HF hospitalizations [12, 14]. Three studies evaluating home visits failed to demonstrate a significant improvement in hospitalization or mortality [13, 17, 18].

**Table 1:** Randomized Trials of Heart Failure Disease Management Programs Using Home Visits

Reference	Duration of intervention	Duration of follow-up	No. of control patients	No. of intervention patients	Primary outcome	Results			
						Primary outcome	ACM	ACH	HFH
Rich et al 1995 [12]	3 months	3 months	140	142	90 days ACH-free survival	0	0	+	+
Jaarsma et al 1999 [13]	1 visit	9 months	95	84	ACH	0	0	0	0
Blue et al 2001 [14]	12 months	12 months	81	84	ACH plus HFH	+	0	0	+
Harrison et al 2002 [15]	2 weeks	5 months	100	92	QOL	+	0	0	0
Stewart and Horowitz 2002 [16]	6 months	6 months	148	149	ACH plus ACM	+	0	0	0
Holland et al 2007 [17]	6 months	6 months	144	149	ACH	0	0	0	NR
Jaarsma et al 2008 [18]	18 months	18 months	348	701	ACM plus HFH	0	0	0	0

ACM: all-cause mortality; ACH: all-cause hospitalization; HFH: heart failure hospitalization; 0: not significant; +: significant; NR: not reported.

In the largest published study to incorporate home visits into the disease management intervention, home visits had no favorable impact on outcomes [18]. The Coordinating Study Evaluating Outcomes of Advising and Counseling in Heart Failure (COACH) randomized 1,023 patients with NYHA class II/III HF to one of three interventions including a control group (n = 339), a basic support group (n = 340), and an intensive support group (n = 344). All three interventions included four visits to a cardiologist over an 18-month follow-up period after an HF hospital discharge. The basic support intervention included nine additional visits to an HF specialist nurse at an outpatient clinic. The intensive support intervention included 18 additional visits to an HF specialist nurse at an outpatient clinic, two home visits by the nurse specialist with one occurring in the first month after discharge, and two multidisciplinary advice sessions. The usual care group included only the four outpatient visits to a cardiologist. The primary endpoint of the composite of HF readmission or all-cause mortality occurred in 141 (42%) control patients, 138 (38%) patients in the basic support group, and 132 (38%) patients in the intensive support group. Analysis of the time to the first event determined hazard ratios of 0.96 (95% CI 0.76 - 1.21; P = 0.73) and 0.93 (95% CI 0.73 - 1.17; P = 0.53) for the composite outcome comparing basic and intensive support against the control group. All-cause mortality and hospitalizations were not different among the patients randomized to the three interventions. The frequency of healthcare contacts initiated by the patient was greater than prescribed in the protocol in all three interventions. This was the greatest in the basic support group where the increase in healthcare contacts was 40% while the increase in the control group was 33%. The increase was only 10% greater than prescribed in the intensive support group.

The most recently published trial including home visits was a randomized comparison against patients who were seen in a walk-in specialty HF clinic. The WHICH (Which Heart Failure Intervention Is Most Cost-Effective & Consumer Friendly in Reducing Hospital Care) study randomized 143 patients to a home-based intervention (HBI) and 137 patients to a specialized HF clinic-based intervention (CBI) with a 12- to 18-month follow-up [19]. The primary outcome was the composite of all-cause unplanned hospitalizations or death. Since there was no control group in this study, conclusions about the relative effectiveness of the either DMP cannot be reached. There was no significant difference in the primary composite outcome between the HBI (71%) and the CBI (76%) (adjusted hazard ratio 0.97; 95% CI 0.73 - 1.30; P = 0.86). There were also no significant differences in unplanned hospitalizations between the HBI (67%) and the CBI (69%) (P = 0.88) or in all-cause mortality between the HBI (22%) and the CBI (28%) (P = 0.25). Patients in the HBI group did have a significantly shorter median duration of days during hospitalizations. The median duration of hospital length of stay with HBI was 4.0 days (interquartile range of 2.0 - 7.0 days) compared to 6.0 days (interquartile range 3.5 - 13 days) with CBI (P = 0.004). Although the HBI was not associated with a significant improvement in the primary outcome compared to the CBI, the shorter hospital stay with HBI was associated with a lower overall healthcare cost (P = 0.03). The costs of providing the patient interventions were not significantly different between HBI ($1,813 per patient) and CBI ($1,829 per patient).

### Outpatient visit interventions

A total of 11 randomized controlled studies meeting eligibility criteria evaluating the efficacy of outpatient clinic visits as part of an HF DMP were identified (Table 2) [18-28]. Two of these trials were previously discussed: the COACH study which found no benefit of frequent visits to a nurse specialist in an outpatient setting compared to usual care and the WHICH study comparing HBI and CBI [18, 19]. Of the remaining nine studies, the primary outcome was significantly improved in five studies [20, 24-27]. However, only two of these studies used hospitalizations or mortality in the primary outcome [26, 27]. Three other studies achieved a statistically significant improvement in their primary outcome [20, 24, 25]. The primary outcomes in these studies were time to readmission, cost-benefit, and cost-utility. All-cause mortality was significantly reduced in two studies, but one of these studies only enrolled a total of 106 patients [25, 26]. Of the seven studies reporting HF-related readmissions, only two significantly reduced those events. The most consistent effect found in the studies utilizing outpatient clinic visits was a significant reduction in all-cause hospitalization which was achieved in five of the nine studies.

**Table 2:** Randomized Trials of Heart Failure Disease Management Programs Using Outpatient Visits

Reference	Duration of intervention	Duration of follow-up	No. of control patients	No. of intervention patients	Primary outcome	Results			
						Primary outcome	ACM	ACH	HFH
Cline et al 1998 [20]	12 months	12 months	110	80	Time to readmission	+	0	0	NR
Ekman et al 1998 [21]	6 months	6 months	79	79	ACH plus ACM	0	0	0	0
Kasper et al 2002 [22]	6 months	6 months	98	102	ACM plus HFH	0	0	0	0
Doughty et al 2002 [23]	12 months	12 months	97	100	ACH plus ACM	0	0	+	0
Ledwidge et al 2002 [24]	3 months	3 months	47	51	Cost benefit	+	0	+	+
Capomolla et al 2002 [25]	12 months	12 months	122	112	Cost utility	+	+	+	NR
Stromberg et al 2003 [26]	12 months	12 months	54	52	ACM plus ACH	+	+	+	0
de la Porte et al 2007 [27]	12 months	12 months	122	118	ACM plus HFH	+	0	+	+
Powell et al 2010 [28]	12 months	31 months	451	451	ACM plus HFH	0	0	0	0

ACM: all-cause mortality; ACH: all-cause hospitalization; HFH: heart failure hospitalization; 0: not significant; +: significant; NR: not reported.

In the largest study using outpatient clinic visits, the Heart Failure Adherence and Retention Trial (HART), 902 patients with NYHA class II/III HF were randomized to one of two interventions [28]. The self-management plus education intervention included 18 two-hour group meetings offered over the first year after randomization. The HF education alone group received 18 “Heart Failure Tip Sheets” mailed on the same schedule as the group meetings. Telephone calls were made within 2 - 3 days after each mailing to ensure receipt and comprehension. Patients were followed for a minimum of 2 years (1 year of treatment and 1 year of post-treatment follow-up). The rate of the primary composite outcome of HF hospitalization plus all-cause mortality was not different in the self-management plus education group (163 events, 40%) compared to the education alone group (171 events, 41%) after a mean follow-up of 2.56 years (odd ratio 0.95; 95% CI 0.72 - 1.26). There were also no significant differences in the secondary endpoints of death, HF hospitalization, all-cause hospitalization, or quality of life.

### Structured telephone support interventions

Disease management interventions relying on outpatient or home visits are resource intensive, costly, and are limited in the numbers of patients that can be impacted. This is especially true for patients in geographically remote areas or those with transportation limitations. Telemanagement using phone calls or the more complex transmission of patient-related clinical data (telemonitoring) over telephone or internet connections have the potential to reach unlimited numbers of HF patients.

A total of 13 randomized controlled studies meeting eligibility criteria evaluating the efficacy of structured telephone support as part of an HF DMP were identified (Table 3) [29-41]. All but two studies used hospitalization or mortality in the primary efficacy outcome [33, 34]. In these two studies, time to hospitalization for HF and medication adherence were the primary outcomes, and neither achieved their primary efficacy endpoint. In the 11 studies using hospitalization, mortality, or both as the primary efficacy endpoint, four studies achieved their primary efficacy endpoint [29, 30, 35, 37]. Two studies were associated with a significant reduction in all-cause mortality, one study was associated with a significant reduction in all-cause hospitalization, and four studies were associated with a significant reduction in HF hospitalizations [29-31, 35-37, 39]. A 2007 meta-analysis which pooled the results of 10 studies of structured telephone support concluded that telephone follow-up significantly reduced HF readmissions, but did not significantly reduce all-cause mortality or all-cause hospitalization [42]. Two of the structured telephone support studies were randomized comparisons against non-invasive telemonitoring DMPs [36, 40]. These studies are discussed further under the non-invasive telemonitoring intervention section.

**Table 3:** Randomized Trials of Heart Failure Disease Management Programs Using Structured Telephone Support

Reference	Duration of intervention	Duration of follow-up	No. of control patients	No. of intervention patients	Primary outcome	Results			
						Primary outcome	ACM	ACH	HFH
Gattis et al 1999 [29]	6 months	6 months	91	90	ACM plus HFH	+	0	NR	+
Riegel et al 2002 [30]	6 months	6 months	228	130	HFH	+	0	0	+
Krumholz et al 2002 [31]	12 months	12 months	44	44	ACH plus ACM	0	0	0	+
Laramee et al 2003 [32]	3 months	3 months	146	141	ACH	0	0	0	0
Tsuyuki et al 2004 [33]	6 months	6 months	136	140	Medication adherence	0	0	0	0
DeBusk et al 2004 [34]	12 months	12 months	234	228	Time to HFH	0	0	0	0
Galbreath et al 2004 [35]	18 months	18 months	359	710	ACM	+	+	0	0
Cleland et al 2005 [36]	8 months	8 months	85	173	ACM plus ACH	0	+	0	0
GESICA Investigators 2005 [37]	16 months	16 months	758	760	ACM plus HFH	+	0	0	+
Riegel et al 2006 [38]	6 months	6 months	65	69	ACH	0	0	0	0
Sisk et al 2006 [39]	12 months	12 months	203	203	ACM plus ACH	0	0	+	0
Mortara et al 2009 [40]	12 months	12 months	160	106	Cardiac death plus HFH	0	0	0	0
DeWalt et al 2012 [41]	12 months	12 months	302	303	ACH plus ACM	0	NR	NR	0

ACM: all-cause mortality; ACH: all-cause hospitalization; HFH: heart failure hospitalization; 0: not significant; +: significant; NR: not reported.

### Non-invasive telemonitoring interventions

A total of 14 randomized controlled studies meeting eligibility criteria evaluating the efficacy of non-invasive telemonitoring support as part of an HF DMP were identified (Table 4) [36, 40, 43-54]. Thirteen of the 14 studies used a primary efficacy endpoint that included hospitalizations, mortality, or both. The lone study that did not include hospitalizations or mortality in the primary outcome used changes in b-type naturetic peptide levels and quality of life [54]. This study did demonstrate a significant improvement in both of the primary endpoints using a mobile-phone-based telemonitoring system.

**Table 4:** Randomized Trials of Heart Failure Disease Management Programs Using Non-Invasive Telemonitoring

Reference	Duration of intervention	Duration at follow-up	No. of control patients	No. of intervention patients	Primary outcome	Results			
						Primary outcome	ACM	ACH	HFH
Goldberg et al 2003 [43]	6 months	6 months	142	138	ACH	0	+	0	NR
Capomolla et al 2004 [44]	12 months	10 months	66	67	ACH plus ACM	+	0	+	+
Cleland et al 2005 [36]	8 months	8 months	85	168	ACH plus ACM	0	+	0	0
Balk et al 2008 [45]	9 months	9 months	113	101	ACH plus ACM	0	0	0	NR
Antonicelli et al 2008 [46]	12 months	12 months	29	28	ACM plus ACH	+	0	+	NR
Soran et al 2008 [47]	6 months	6 months	155	160	CV death plus HFH	0	0	0	0
Woodend et al 2008 [48]	3 months	12 months	59	62	ACH	0	NR	0	NR
Dar et al 2009 [49]	6 months	6 months	91	91	ACH plus ACM	0	NR	0	0
Giordano et al 2009 [50]	12 months	12 months	230	230	ACH plus CV mortality	NR	0	+	+
Mortara et al 2009 [40]	12 months	12 months	160	195	CV death plus HFH	0	0	0	0
Weintraub et al 2010 [51]	3 months	3 months	93	95	HFH	+	0	0	+
Chaudhry et al 2010 [52]	6 months	6 months	827	826	ACM plus ACH	0	0	0	0
Koehler et al 2011 [53]	26 months	26 months	356	354	ACM	0	0	0	0
Seto et al 2012 [54]	6 months	6 months	50	50	Changes in BNP/QOL	+	0	0	0

ACM: all-cause mortality; ACH: all cause hospitalization; HFH: heart failure hospitalization; CV: cardiovascular; 0: not significant; +: significant; NR: not reported; BNP: b-type naturetic peptide; QOL: quality of life.

Of the remaining 13 studies, three achieved their primary efficacy endpoint [44, 46, 51]. Twelve of the 13 studies reported the effect of the DMP on cardiac or all-cause mortality with only two studies demonstrating a significantly positive effect on this outcome [36, 43]. Three studies significantly reduced all-cause hospitalizations [44, 46, 50]. Ten of the 14 studies reported rates of HF hospitalizations with three of the 10 studies demonstrating significant reductions in these hospitalizations [44, 50, 51].

A Cochrane database review conducted a meta-analysis published in 2010 which included a total of 27 controlled studies including 11 studies using non-invasive telemonitoring (2,710 patients) and 16 studies using structured telephone support (5,613 patients) [55]. All-cause mortality was significantly reduced by non-invasive telemonitoring (RR 0.66; 95% CI 0.54 - 0.81; P < 0.001). While structured telephone support reduced all-cause mortality, the effect was not statistically significant (RR 0.88; 95% CI 0.76 - 1.01; P = 0.08). HF hospitalizations were significantly reduced by both telemonitoring (RR 0.79; 95% CI 0.67 - 0.94; P = 0.008) and structured telephone support (RR 0.77; 95% CI 0.68 - 0.87; P < 0.0001).

There were two randomized controlled studies comparing structured telephone support against non-invasive telemonitoring. The Trans-European Network-Home Care Management System (TEN-HMS) study randomized 426 patients to usual care (n = 85), structured telephone support (n = 173), or to non-invasive telemonitoring (n = 168) [36]. Telemonitoring included twice daily transmission of weight, blood pressure, heart rate, and cardiac rhythm. The primary endpoints of all-cause mortality plus all-cause hospitalization as well as all-cause and HF hospitalizations were not different between either of the intervention groups compared to usual care. The differences in these endpoints were also not significant between telephone support and telemonitoring. However, both intervention groups were associated with significant reductions in all-cause mortality compared to usual care.

The second randomized trial comparing structured telephone support and telemonitoring randomized 160 patients to usual care and 301 patients to one of three intervention groups [40]. Strategy 1 employed structured telephone support alone (n = 104), strategy 2 employed structured telephone support plus weekly transmission of vital signs including changes in weight, blood pressure and symptoms (n = 96), and strategy 3 employed the same intervention used in strategy 2 plus a monthly 24-h cardiorespiratory recording (n = 101). The cardiorespiratory recording included 24-h continuous electrocardiographic monitoring and physical activity. All-cause hospitalization, HF hospitalization, and mortality were not significantly reduced in the more intensive strategy 2 and 3 intervention groups compared to strategy 1 patients.

### Invasive telemonitoring interventions

Four different types of invasive hemodynamic monitoring interventions have been evaluated in patients with HF [56]. These include intrathoracic impedance monitoring, pulmonary artery pressure monitoring, right ventricular pressure monitoring, and left atrial pressure monitoring. There are relatively few randomized, controlled trials using invasive hemodynamic monitoring for the prevention of hospital readmission in patients with HF (Table 5).

**Table 5:** Randomized Trials of Heart Failure Disease Management Programs Using Invasive Hemodynamic Monitoring

Reference	Type of hemodynamic monitoring	Duration of intervention	Duration of follow-up	No. of control patients	No. of intervention patients	Primary outcome	Results			
							Primary outcome	ACM	ACH	HFH
Van Veldhuisen et al 2011 [58]	Intrathoracic impedance	14.5 months	14.5 months	167	168	ACM plus HFH	0	0	0	0
Crossley et al 2011 [62]	Intrathoracic Impedance	15 months	15 months	983	1014	Time to clinical decision	+	0	0	0
Landolina et al 2012 [63]	Intrathoracic impedance	16 months	16 months	101	99	ED and urgent OPV	+	NR	0	0
Abraham et al 2011 [64]	Pulmonary artery pressures	15 months	15 months	270	280	HFH	+	0	0	+
Bourge et al 2008 [66]	Right ventricular pressures	6 months	6 months	140	134	HF-related urgent events	0	NR	NR	0
Adamson et al 2011 [67]	Right ventricular pressures	12 months	12 months	198	202	HF-related urgent events	0	NR	0	0

ACM: all-cause mortality; ACH: all-cause hospitalization; HFH: heart failure hospitalization; 0: not significant; +: significant; NR: not reported; ED: emergency department; OPV: outpatient visits.

The largest numbers of studies published to date evaluated intrathoracic impedance monitoring with or without the addition of other physiologic variables. Many patients with severe HF have indications for implantable cardioverter defibrillator (ICD) or cardiac resynchronization therapy with defibrillator (CRT-D) therapy [56]. Intrathoracic impedance monitoring is calculated using an algorithm (OptiVol, Medtronic, Minneapolis, MN, USA) that performs a series of electrical impedance measurements between the ICD or CRT-D device case and the pacing electrode located in the right ventricle. A characteristic of that electrical current is impedance, or the resistance the electrical signal experiences as it passes from the device to the electrode [57]. Impedance decreases in water as electricity is conducted with less resistance in water than in air. When patients develop fluid accumulation in the lung and pulmonary vasculature due to worsening HF, the impedance in the chest cavity declines and the device can measure that change in impedance [57]. The ICD or CRT-D can also provide additional information such as heart rate variability, patient activity, presence of arrhythmias, delivery of shock therapy, and device integrity such as lead malfunction [56]. This information can be relayed automatically to the clinician using remote wireless technology. Outside of the United States an audible patient alert can also be triggered by changes in impedance [58]. Several studies have been able to demonstrate that changes in intrathoracic impedance with or without the additional device detected information can predict HF decompensation and hospitalization for HF [57, 59-61].

There have been three randomized controlled trials using intrathoracic impedance in addition to other device derived parameters in patients with HF [58, 62, 63]. The Diagnostic Outcome Trial in Heart Failure (DOT-HF) randomized 335 patients with HF (96% NYHA class II/III) who had received ICD or CRT-D therapy to a control group (n = 167) or to a remote access group with an audible patient alert (n = 168) [58]. Over an average 15-month follow-up, access to device derived parameters including intrathoracic impedance and the audible patient alert was associated with more HF hospitalizations (HR 1.79; 95% CI 1.08 - 2.37; P = 0.022) and three times as many outpatient visits (P < 0.0001). The number of deaths was not significantly different between the treatment groups.

The Clinical Evaluation of Remote Notification to Reduce Time to Clinical Decision Trial (CONNECT) randomized 1,997 HF patients implanted with an ICD or CRT-D to automatic clinician alerts using a wireless platform (Medtronic CareLink Network) (n = 1,014) or to in-office device interrogation (n = 983) [62]. The primary study endpoint was the time from a predefined clinical event to the time a clinical decision was made. Clinical events included changes in intrathoracic impedance as well as arrhythmia events and device/lead integrity alerts. A major study limitation was that 43% of the clinical events were not automatically transmitted to the clinician due to the alert programming being turned off or not having been reset after a prior alert. Although the remote automatic clinician alert reduced the median time to decision from 22 days in the office monitoring group to just under 5 days in the remote access group, the automatic alert did not reduce hospitalizations, office visits, or mortality.

The Evolution of Management Strategies of Heart Failure Patients with Implantable Defibrillators (EVOLVO) study randomized 200 patients with HF and an ICD/CRT-D to remote monitoring using the Medtronic CareLink wireless feature with intrathoracic impedance and other device alerts (n = 98) or to office follow-up (n = 101) [63]. In the office follow-up treatment group, remote automatic clinician alerts were programmed off, but audible patient alerts were turned on. In the remote monitoring group, the audible alert was programmed off. The primary study endpoint was the rate of emergency department or urgent in-office visits for HF, arrhythmias, or ICD alerts. At the end of 16 months, 75 events occurred in the remote group compared to 117 in the in-office group (RR 0.65; 95% CI 0.49 - 0.88; P = 0.005). This significant difference resulted from a reduction in visits for HF (48 vs. 92 visits). Visits for arrhythmias and ICD alerts were not different between the two groups. There were also no significant differences in all-cause or HF hospitalizations. The time to clinical decision in this study was approximately 1.5 days in the remote access group and 25 days in the in-office group.

There has been one randomized trial evaluating pulmonary artery pressure monitoring using a wireless, passive, radiofrequency sensor implanted into a distal branch of the descending pulmonary artery [64]. The CardioMEMS Heart Sensor Allows Monitoring of Pressure to Improve Outcomes in NYHA class III Heart Failure Patients (CHAMPION) study randomized 550 patients with the wireless pressure monitor to a treatment group in which clinicians were given access to the pressure results (n = 270) or to a control group in which clinicians did not receive pressure results (n = 280). The primary study endpoint was HF hospitalizations at 6 months. The rate of HF hospitalizations was significantly reduced in the treatment group at 6 months and at the end of the entire follow-up period (15 months). At 6 months, there were 84 HF hospitalizations in the treatment group and 120 in the control group (28% RRR; P = 0.0002). At 15 months, there was a 37% reduction in HF hospitalizations in the treatment group compared to the control group (P < 0.0001). All-cause hospitalization and mortality were not reported.

Despite the favorable outcome of the CHAMPION study, a Food and Drug Administration (FDA) Advisory Panel initially recommended against approval of the CardioMEMS HF device in 2011 [65]. There was concern that a wider disparity in the distribution of HF hospitalizations occurred with a substantial proportion of patients not being hospitalized. The Advisory Panel raised concerns that the observed variance was larger than the observed means. Another major concern was that the treatment group received excessive treatment support from investigators who had frequent communications with physicians caring for patients in the treatment group but not in the control group. Following a second FDA Advisory Panel meeting in 2013 during which a post-marketing efficacy and safety evaluation program was recommended, the FDA approved the CardioMEMS HF System - P100045^TM^ in May 2014. This system was approved for implant in NYHA class III HF patients who have been hospitalized for HF in the previous year. The device has coverage for inpatient reimbursement through the Center for Medicare and Medicaid Services.

There were two randomized, controlled trials evaluating the benefit of right ventricular pressure monitoring in patients with HF [66, 67]. The Chronicle Offers Management to Patients with Advanced Signs and Symptoms of Heart Failure (COMPASS-HF) [66]. This study randomized 274 NYHA class III/IV HF patients who had an implantable continuous hemodynamic monitor (ICHM) placed in the right ventricular outflow tract or right ventricular septum. This sensor (Chronicle) detects heart rate, body temperature, patient activity, right ventricular systolic and diastolic pressures, and changes in those pressures over time. After implantation, patients were randomized to an intervention group in which physicians could review the ICHM information on a weekly basis (n = 134) or to a control group in which that data were not available (n = 140). After 6 months, ICHM data were made available for both groups of patients. The primary outcome was the frequency of HF-related events (hospitalizations, emergency department visits, urgent outpatient visits) at 6 months of follow-up. During that follow-up period, 84 HF events occurred in 44 patients in the intervention group and 113 events occurred in 60 patients in the control group. This 21% relative risk reduction failed to reach statistical significance (P = 0.33). All-cause hospitalizations and mortality were not reported.

The Reducing Decompensation Events Utilizing Intracardiac Pressures in Patients with Chronic Heart Failure (REDUCEhf) randomized 400 patients who had the right ventricular pressure monitor (Chronicle) implanted to a treatment group in which pressure data were available (n = 202) or to a control group where pressure data were not made available (n = 198) [67]. The primary outcome was a composite of HF hospitalizations, emergency department visits, or urgent clinic visits over a 12-month follow-up. The intervention failed to have any effect on these outcomes with 91 events occurring in 43 patients in the treatment group compared to 90 events in 43 patients in the control group (P = 0.98). Mortality was not reported.

There have been no randomized, controlled studies using left atrial pressure monitoring. The Hemodynamically Guided Home Self-Therapy in Severe Heart Failure Patients (HOMEOSTASIS) followed 40 NYHA class III/IV patients for a median of 25 months after implantation of a left atrial pressure monitor (HeartPOD, St. Jude Medical, St. Paul, MN, USA) [68]. Patients had significant improvements in functional class, reductions in left ventricular pressures, and fewer substantial increases in left atrial pressure. These favorable outcomes most likely resulted from more efficient use of diuretic and vasodilator therapy in response to changes in left atrial pressure.

## Discussion

The Heart Failure Society of America and the European Society of Cardiology Heart Failure Association recommend enrollment in DMPs for patients with HF who have been recently hospitalized or for high-risk HF patients [69, 70]. High-risk patients include those with renal dysfunction, diabetes mellitus, chronic obstructive pulmonary disease, New York Heart Association (NYHA) class III or IV symptoms, frequent hospitalizations for any reason, multiple comorbidities, a history of depression, cognitive impairment, inadequate social or home support, poor health literacy, or a history of non-adherence to treatment recommendations.

The recommended elements of an HF DMP are summarized in Table 6. Although comprehensive discharge planning with post-discharge support has been shown to reduce readmission rates in HF patients, substantial numbers of patients continue to be readmitted [71, 72]. Recent changes in healthcare policy and the HRRP have increased the importance of reducing hospital readmissions in patients discharged with an HF diagnosis [73]. The challenges associated with impacting HF readmissions are enormous. With more than half of readmissions for reasons other than HF, DMPs specifically directed at HF alone would be expected to fail to reduce readmissions in a large number of patients.

**Table 6:** HFSA Recommended Elements of Heart Failure Disease Management Programs [69]

1	Comprehensive education and counseling individualized to the patient and patients’ environment
2	Promotion of self-care behaviors including potentially self-titration of diuretic dosing (with family member/healthcare provider assistance)
3	Emphasis on behavioral strategies to ensure adequate compliance
4	Adequate follow-up after hospital discharge or clinical instability (preferably within the first 7 days after event)
5	Optimization of oral therapy especially evidence-based therapy
6	Increased access to healthcare providers
7	Early attention to signs and symptoms of fluid overload
8	Assistance with financial and social concerns

HFSA: Heart Failure Society of America.

The results of our systematic review, limited to randomized, controlled trials, found substantial heterogeneity in the results of all of the available types of HF DMPs. The vast majority of the published studies were not adequately powered to demonstrate reductions in clinical endpoints. Programs that utilized face-to-face interventions either in outpatient clinics or at patients’ homes were able to demonstrate significant reductions in HF hospitalizations in just four of 13 studies reporting that outcome [12, 14, 23, 27]. Home visits appear to be less effective in reducing all-cause hospitalizations compared to outpatient visits. Neither approach had a consistent impact on mortality compared to usual care. There is only a single randomized comparison of these two treatment interventions [19]. As this study failed to show a significant difference between the interventions, it is impossible to reach valid conclusions concerning their relative effectiveness. It is plausible to consider that office-based interventions may be able to provide a wider range of diagnostic and treatment options (i.e. chest X-ray, echocardiograms, etc.) that may have accounted for the more consistent impact of this intervention on all-cause hospitalization. Neither of these interventions would be considered inexpensive.

The results of the COACH and HART studies indicate that intensive face-to-face interventions are no better than less costly and less time-intensive interventions in patients with mild-to-moderate HF [18, 28]. The study populations were similar in that the vast majority of patients had NYHA class II/III HF and were receiving evidence-based therapies (ACEI 83-85% and beta-blockers 66-70%). Patients in COACH were probably a higher risk population as only patients discharged from the hospital following an admission for HF were enrolled while patients in HART were recruited from both the hospital and outpatient clinic. Neither study was able to determine why the more intensive intervention failed to produce benefit particularly when compared to earlier, smaller studies. One explanation may be that a higher proportion of control patients in COACH and HART were receiving evidence-based therapies and that current levels of expertise provided through “usual care” are substantially improved compared to patients treated with “usual care” in the early 1990s. Another possible explanation is that more intensive face-to-face interventions used in COACH and HART are actually not effective. Earlier studies demonstrating benefit of face-to-face healthcare provider and patient interactions included smaller numbers of patients generally treated at a single site [12]. These studies may have overestimated the benefit of such interactions. The conclusions of the investigators of both the COACH and HART are that HF DMPs should not be abandoned, but that further research is needed to better define what elements of such programs are effective and how they should be implemented. It would be erroneous to assume that one type of DMP will fit all types of HF patients or all healthcare systems across the three phase terrain of readmissions.

DMPs relying on telephone or non-invasive telemonitoring have the advantage of being able to reach large numbers of patients who live in geographically distant areas or who have other reasons for limited travel. In addition, structured telephone support should be a relatively inexpensive treatment option. Non-invasive telemonitoring is associated with greater expense and requires a certain degree of health-literacy on the part of patients who must interact with the system that transmits patient information to the healthcare provider.

The results of structured telephone support and non-invasive telemonitoring have also been heterogeneous. Only four of the 13 studies evaluating structured telephone support were able to demonstrate reductions in HF hospitalizations [29-31, 37]. All-cause hospitalizations were reduced in one structured telephone support study while all-cause mortality was reduced in two of these studies [35, 36, 39]. The largest of the structured telephone support studies was able to demonstrate a statistically significant reduction in the primary composite outcome of all-cause mortality plus HF hospitalizations primarily due to a significant reduction in HF hospitalizations [37].

Of the 14 randomized, controlled trials of non-invasive telemonitoring, only 10 reported on rates of HF readmissions. In these 10 studies, HF hospitalizations were reduced in only three [44, 50, 51]. All-cause hospitalization was reported in all 14 studies with three reporting significant reductions [44, 46, 50]. Two of the 12 studies reporting mortality were able to demonstrate significant reductions in all-cause mortality [36, 43].

The Cochrane Library meta-analysis of 27 randomized controlled trials of structured telephone support compared with non-invasive telemonitoring found significant reductions in HF hospitalization for both interventions [55]. In addition, this meta-analysis found a significant reduction in all-cause mortality with telemonitoring and trend to a significant reduction with structured telephone support. However, the results of meta-analyses are generally only considered to be hypothesis generating. In addition, both randomized comparisons of structured telephone support and non-invasive telemonitoring failed to demonstrate one intervention to be superior to the other or to less intensive interventions [36, 40].

The value of invasive hemodynamic monitoring as a part of an integrated disease management strategy for HF patients remains an area of intense research interest. Of the available published invasive hemodynamic monitoring studies, the CHAMPION study generated the most interest due to the favorable reduction in HF hospitalization [64]. With the recent FDA approval of the CardioMEMS HF System, the clinical utility of this device will be closely followed to determine if it performs as well in general clinical use as it did in the CHAMPION study.

The largest volume of published data is with intrathoracic impedance monitoring typically used in combination with a variety of other device derived parameters. This approach is limited to patients who qualify for insertion of an ICD or CRT-D. In two of the three controlled trials using intrathoracic impedance monitoring, the primary outcome was time to clinical decision or a reduction in urgent emergency department or clinic visits [62, 63]. Although potentially clinically relevant, achievement of this primary outcome appears to be irrelevant considering that neither of those trials was able to demonstrate reductions in hospitalizations or mortality. It should also be noted that only one of the intrathoracic impedance studies was adequately powered to evaluate clinical events which were not favorably impacted [62].

Both of the studies assessing right ventricular pressure indices also failed to reduce urgent HF-related healthcare contacts including hospitalization [66, 67]. Neither study reported the impact of monitoring on mortality. Both of these studies were underpowered for clinical events. The REDUCEhf was stopped prematurely by the manufacturer due to a high rate of lead failures in other studies in which that particular lead was used [67]. As a result, only 400 of a planned 1,350 patients were actually enrolled in the trial.

Although right ventricular pressure (CHRONICLE) and pulmonary artery pressure (CardioMEMS HF System) monitoring require implantation of a special sensor that does not offer the other therapeutic features of an ICD/CRT-D, there is no waiting period for pressure monitoring to start. With ICD/CRT-D therapy, intrathoracic impedance monitoring requires a waiting period of about 30 days before monitoring is considered reliable. Additional studies with the other types of invasive hemodynamic monitoring will be required before they can be considered a standard of care for the patient with severe HF.

## Summary and Conclusion

The currently available evidence supporting the efficacy of HF DMPs based on our systematic review restricted to randomized, controlled trials, demonstrated highly inconsistent results. This should not be interpreted to indicate that HF DMPs are not potentially effective. Rather, our data suggest that one approach applied to a broad spectrum of different patient types may not be effective. HF DMPs should be flexible enough to be individualized to meet the needs of the specific patient. An effective HF DMP remains as much an art as it does science.
